# Fatigue Among Caregivers of Hospitalized Patients

**DOI:** 10.3390/clinpract16010022

**Published:** 2026-01-22

**Authors:** Eleni Maria Mitrou, Lamprini Avramopoulou, Dimitrios Alefragkis, Athanasia Tsami, Maria Polikandrioti

**Affiliations:** 1 MSc in Applied Clinical Nursing, Nursing Department, University of West Attica, 12243 Athens, Greece; 2 Nursing Department, University of West Attica, 12243 Athens, Greece

**Keywords:** fatigue, insomnia, anxiety, caregivers, hospitalized patients

## Abstract

**Introduction**: Caregiving has been an emerging public health priority mainly due to the rapid pace of population aging, increase in chronic diseases and shortages of health professionals. In clinical settings, caregivers have a crucial role by providing support to patients. Consequently, they may experience physical and emotional burden mainly attributed to environmental, personal or family stressors. The aim of this study was to evaluate fatigue and the associated factors among caregivers of hospitalized patients in medical-surgical wards. **Methods and Material**: In the present study caregivers of hospitalized patients in medical and surgical wards were enrolled. Collection of data was performed with the following: a. Fatigue Assessment Scale (FAS), b. Zung Self-Rating Anxiety Scale (SAS) and c. Athens Insomnia Scale (AIS), which included patients’ characteristics. In this cross-sectional study, participants were selected using the method of convenience sampling. **Results**: Of the 142 participants, the majority were spouses (64.8%), female (64.8%) and over 60 years old (53.6%). The mean FAS score was 25.9 ± 9.3, the mean SAS was 38.1 ± 9.0, and the mean AIS score was 7.6 ± 4.7, indicating moderate, moderate to low and moderate levels of fatigue, anxiety and insomnia, respectively. Moreover, fatigue showed a positive linear correlation with both anxiety (Spearman’s rho = 0.713) and insomnia (Spearman’s rho = 0.671). The factors found to be statistically significantly associated with caregivers’ fatigue were the following: gender (*p* = 0.001), length of hospitalization (*p* = 0.013), experience of environmental stressors (*p* = 0.045), experience of financial stressors (*p* = 0.001), and unfamiliarity with the provision of care (*p* = 0.001). **Conclusions**: Provided that caregivers’ involvement in care not only enhances patient well-being but also supports clinical teams, it is widely comprehended that addressing their needs should be emphasized.

## 1. Introduction

In recent decades, demographic shifts and the rising prevalence of chronic diseases have contributed to a substantial increase in the number of older adults and multi-morbid patients presenting to Emergency Departments and subsequently requiring hospital admission. This trend has imposed significant challenges on healthcare systems, contributing to hospital overcrowding [1]. In addition to this complexity, persistent staffing shortages in Greek hospitals have resulted in diminished patient-to-nurse ratios, thereby creating an expectation that hospitalized patients be accompanied by informal caregivers who assist in meeting their needs [2]. Moreover, the traditional structure of Greek society, characterized by strong familial bonds, continues to serve as a fundamental source of support for patients [2].

Caregiving needs are rising worldwide. For instance, in Europe, projections suggest that, by 2030, one in four workers will take on caregiving responsibilities with 41.8 million people already providing unpaid care to adults over 50 [3]. In the United States, demographic shifts point in the same direction, with an estimated 73 million Americans aged 65 or older expected to require daily assistance by 2030 [4].

Informal caregivers are individuals who provide unpaid support and medical assistance to people with illnesses, disabilities, or chronic conditions [5,6]. However, the extent of their involvement varies considerably and is influenced by factors such as the severity of the patient’s condition and the family’s perceived level of helplessness and social isolation [6,7,8]. Hospitalization reflects a deterioration in patients’ health status, which can be inherently stressful; consequently, caring for a hospitalized individual imposes a substantial burden on caregivers [7]. In the context of hospitalized patients, the caregiver’s role is indispensable in monitoring patient status, managing complex care needs, delivering primary bedside care and preventing adverse events across both medical and surgical wards [2]. Caregivers of hospitalized patients often experience fatigue, encompassing both physical and mental dimensions, attributable to the extended hours spent at the patient’s bedside combined with the demands of their ongoing daily responsibilities [5,6].

The Theory of Unpleasant Symptoms conceptualizes fatigue as a multidimensional symptom characterized by its timing, intensity, quality, and distress. Fatigue arises from the complex interplay of physiological, psychological, and situational factors, which collectively influence its manifestation and impact on functional performance. The theory further posits that fatigue, like other symptoms, affects an individual’s physical and cognitive abilities, thereby influencing overall outcomes and potentially interacting with other concurrent symptoms [9]. In parallel, the Stress Process Model frames fatigue as a primary stressor, particularly in demanding roles such as caregiving, where it results directly from the burdens of tasks and responsibilities [10]. Although fatigue typically ameliorates with rest and does not significantly impair daily functioning, it may become pathological when persisting beyond six months, thereby adversely impacting quality of life, emotional and social functioning, and the capacity to perform routine activities [7].

Fatigue varies across several contexts, with its underlying medical etiology remaining unidentified in approximately 59–64% of adults reporting fatigue. Due to its nonspecific nature, the differential diagnosis and management of fatigue pose considerable challenges. Moreover, both patients and clinicians often underestimate fatigue, frequently attributing it to everyday stress rather than recognizing it as a potential indicator of underlying medical conditions [11,12].

Measuring fatigue among caregivers is essential because it directly affects their health, well-being, and ability to provide care. Identifying fatigue early helps target interventions to improve both caregiver and care recipient outcomes. Accurate assessment also informs policies to better support caregiver needs.

The aim of this study was to evaluate fatigue and the associated factors (anxiety, insomnia, participant characteristics) among caregivers of hospitalized patients in medical-surgical wards.

## 2. Materialand Methods

### 2.1. Study Design, Participants

In this study 142 caregivers of hospitalized patients in a general public hospital in Attica were enrolled from February 2025 to September 2025. The sample studied was selected using the method of convenience sampling.

In terms of participant recruitment, caregiver relationship to the care recipient was ascertained through a structured screening process, wherein participants were asked to specify their familial or relational connection. This information was collected via the questionnaire, which included predefined categories such as spouse and child. A total of 150 potential participants were approached, of whom 142 consented to participate. The remaining eight individuals declined participation.

In this observational cross-sectional study, participants were assessed at a single point in time, and the primary aim was to explore relationships between variables; therefore, no formal sample size calculation was performed. This can be justified because this study was mainly descriptive and exploratory in nature, aiming to describe trends, identify potential relationships, and generate hypotheses for future research [13].

### 2.2. Inclusion and Exclusion Criteria of the Sample

Informal caregivers are defined as the family members who provide unpaid support and assistance to patients [5,6]. Therefore, in this study only spouses or children of hospitalized patients were included. The criteria for inclusion in this study were the following abilities: (i) to write, read, and comprehend Greek language; (ii) to read and sign the informed consent form and (iii) caregivers of hospitalized patients solely in medical-surgical wards as the type of ailment or clinical environment could influence their experience of fatigue.

Exclusion criteria were caregivers of hospitalized patients already diagnosed with the following: (i) mental disorders, (ii) cognitive impairment and (iii) caregivers of hospitalized patients in intensive care units (ICUs) for the reason that they face greater challenges due to the critical condition of the patients and the associated heightened care needs. Also, other informal caregivers such as friends, distant relatives, or neighbors who often provide substantial support were excluded to maintain a focused and homogenous sample of primary family caregivers. Additionally, caregivers under 18 years of age were excluded from this study due to ethical considerations related to consent and assent, as well as potential developmental differences that may influence caregiving roles. Caregiving responsibilities among individuals below 18 years often differ in scope and nature compared to adult caregivers, and their inclusion could introduce variability and confound this study’s focus on adult caregiving dynamics.

### 2.3. Data Collection

Data were collected through face-to-face interviews conducted during evening shifts, when caregivers were available and relieved of patient care responsibilities. Completion of the questionnaire required approximately 20 to 30 min and was conducted in a private office to ensure confidentiality. The interviews conducted were structured, consisting of a fixed set of predetermined questions with no open-ended items. This approach ensured consistency across all participants and facilitated quantitative analysis of responses.

### 2.4. Research Instrument

Collection of data was performed using the following: a. Fatigue Assessment Scale (FAS), b. Self-Rating Anxiety Scale (SAS-Zung) and c. Athens Insomnia Scale, which included patients’ characteristics.

#### 2.4.1. Assessment of Caregivers’ Fatigue

The Fatigue Assessment Scale (FAS) was used to assess caregivers’ fatigue. The FAS is an internationally accepted self-report instrument designed to measure generalized fatigue in both clinical and research populations. It consists of 10 items covering physical and mental aspects of fatigue. Responses are rated on a five-point Likert scale ranging from “never” to “always.” The total score ranges from 10 to 50, with higher scores indicating greater fatigue severity. Some items require reverse scoring to ensure accurate interpretation of the overall score. The FAS has been translated into various languages and applied across diverse cultural contexts, demonstrating satisfactory reliability and validity, which makes it particularly useful for monitoring fatigue. More specifically, the FAS, as validated by Alikari et al. [14], reported a Cronbach’s alpha of 0.88, indicating good internal consistency reliability.

#### 2.4.2. Assessment of Caregivers’ Anxiety

Caregivers’ anxiety was assessed using the Zung Self-Rating Anxiety Scale (SAS), an internationally recognized psychometric instrument designed to evaluate both the psychological and somatic manifestations of anxiety. The scale comprises 20 items reflecting the respondent’s experiences during the past week. Responses are rated on a four-point Likert scale (1–4), indicating the frequency of symptoms. Certain items are reverse-scored to ensure accuracy in the overall score. The final score is obtained by summing the item responses, providing an overall estimate of anxiety, with higher scores indicating greater symptom severity. The scale has been validated across diverse populations, demonstrating satisfactory reliability and validity, which supports its use in both research and clinical settings. The Greek version of the Zung Self-Rating Anxiety Scale has demonstrated good reliability, with a reported Cronbach’s alpha of 0.897, confirming its internal consistency in Greek-speaking populations [15].

#### 2.4.3. Assessment of Caregivers’ Insomnia

Symptoms of insomnia were assessed using the Athens Insomnia Scale (AIS), a psychometric instrument developed for the quantitative evaluation of insomnia in accordance with the diagnostic criteria of the ICD-10. The scale comprises eight items: the first five assess difficulties related to sleep initiation and maintenance, early morning awakening, and total sleep duration, while the final three address daytime functioning and well-being. Each item is scored on a four-point scale (0–3), yielding a total score ranging from 0 to 24. Higher scores indicate greater severity of insomnia symptoms, with a score of ≥6 proposed as the indicative threshold for diagnosing insomnia. The scale has been translated and utilized across diverse populations, demonstrating high reliability and validity, thereby confirming its applicability in both clinical and research settings. The AIS was developed and validated by Soldatos et al. [16], who reported excellent internal consistency in their original validation study, with a Cronbach’s alpha of 0.89.

### 2.5. Ethical Considerations

The study protocol received approval from the Research Committee of the public hospital (No. 38242/14.11.2024) where the research took place. Participants were informed about this study’s objectives by the researcher, and written informed consent was obtained from all participants. Data collection procedures ensured anonymity and confidentiality. All participants were made aware of their right to refuse participation or withdraw from this study at any time, in accordance with the ethical principles outlined in the Declaration of Helsinki (1989) by the World Medical Association.

### 2.6. Statistical Analysis

Qualitative data are presented as absolute and relative (%) frequencies, while quantitative data are summarized using mean, standard deviation, median, and interquartile range (IQR). Normality of distributions was assessed using the Kolmogorov–Smirnov test and graphically through histograms. The Kruskal–Wallis and Mann–Whitney tests were applied to examine associations between fatigue scores and caregivers’ characteristics. Spearman’s rho correlation coefficient was used to evaluate linear relationships between fatigue, anxiety, and insomnia among caregivers. Multiple linear regression analysis was performed to assess the effect of caregivers’ characteristics on their fatigue levels. Results are reported with β regression coefficients and 95% confidence intervals (CIs). A significance level of 5% was considered statistically significant. All analyses were conducted using SPSS version 25 (SPSS Inc., Chicago, IL, USA).

## 3. Results

### 3.1. Sample Description

Table 1 presents the demographic characteristics of caregivers in relation to the hospitalized patients they cared for. The majority were spouses (64.8%), female (64.8%), over 60 years old (53.6%), married (63.4%), of tertiary education level (40.8%), employees (73.3%), residents of Attica (47.2%), while their loved person had hospital duration of 4–6 days (28.9%).

Table 2 presents perceptions of caregivers in relation to the hospitalized patients they cared for. The majority were sufficiently informed about the patient’s health status (47.9%), they declared to provide emotional support to the patient after hospital discharge (57.1%), experienced environmental stressors (78.2%), financial stressors due to the patient’s illness (28.9%), stated unfamiliarity with care provision (36.6%), declared the need for communication with nurses (92.3%).

### 3.2. Measurement Fatigue, Anxiety, Insomnia

Table 3 presents the results concerning caregivers’ anxiety, fatigue, and insomnia. The three scales used demonstrated satisfactory to very good internal consistency, as indicated by Cronbach’s alpha values. Specifically, Cronbach’s α was 0.864 for the Zung Anxiety Scale, 0.730 for the FAS scale, and 0.881 for the AIS insomnia scale.

Regarding fatigue, based on the FAS (range 10–50), the mean score was 25.9 with a standard deviation of 9.3. The median was 25, with an interquartile range (IQR) of 19–31. This indicates that half of the caregivers had fatigue scores below 25, and half had scores within the range of 19–31. These values suggest that caregivers experience moderate fatigue.

As for anxiety, the mean score on the Zung scale (range 20–80) was 38.1 with a standard deviation of 9.0, while the median was 37 with an IQR of 32–45. This suggests that half of the caregivers had anxiety scores below 37, and half within the range of 32–45. These findings indicate that most caregivers exhibit low to moderate anxiety levels.

Concerning insomnia, the AIS (range 0–24) recorded a mean score of 7.6 with a standard deviation of 4.7, while the median was 7 with an IQR of 4–10. This suggests that half of the caregivers had insomnia scores below 7, and half within the range of 4–10. These values reflect a moderate level of insomnia within the sample. Notably, 64.8% of caregivers had an AIS score ≥ 6, the indicative threshold for clinically significant sleep problems.

### 3.3. Association Between Scales

Table 4 presents the correlation between the scales. Fatigue, anxiety, and insomnia among caregivers were found to be mutually correlated, all with *p* = 0.001. Specifically, fatigue showed a very strong positive linear correlation with both anxiety (Spearman’s rho = 0.713) and insomnia (Spearman’s rho = 0.671), indicating that, as caregivers’ levels of anxiety and insomnia increase, their levels of fatigue also increase.

### 3.4. Association of Fatigue with Caregivers’ Characteristics

Table 5 presents the association between caregivers’ fatigue and their characteristics. The factors found to be statistically significantly associated with caregivers’ fatigue levels were: gender (*p* = 0.001), length of hospitalization (*p* = 0.013), experience of environmental stressors (*p* = 0.045), of financial stressors (*p* = 0.001), and unfamiliarity about provision of care (*p* = 0.001).

More specifically, women reported higher fatigue (median = 26, IQR = 21–33) compared with men (median = 21, IQR = 16–26), indicating that female caregivers experienced significantly greater fatigue. Fatigue increased with the length of hospitalization. The lowest levels were observed in the 4–6-day group (median = 21, IQR = 17–26), while the highest levels were recorded for hospitalizations exceeding 10 days (median = 27, IQR = 19–35). Similarly, for 7–10 days, the median was 27 (IQR = 21–32), highlighting that prolonged hospitalization is associated with greater fatigue. Caregivers who experienced environmental stressors reported higher fatigue (median = 24, IQR = 19–32) compared with those who responded negatively (median = 21, IQR = 17–26). The highest fatigue levels were observed among caregivers with severe financial stressors (median = 29, IQR = 23–33), while those with no financial worries had the lowest (median = 18.5, IQR = 15–24). The intermediate categories (“quite a lot” and “a little”) showed median values around 23 (IQR ≈ 19–32). Fatigue was also markedly higher among caregivers expressing unfamiliarity about provision of care (median = 28, IQR = 24–32). In contrast, those with little or no unfamiliarity had lower scores (median = 19, IQR = 16–22).

### 3.5. Estimation of the Impact of Caregivers’ Characteristics on Fatigue (FAS)

A multiple linear regression analysis was conducted to estimate the impact of caregivers’ characteristics (independent variables) on their experienced fatigue (FAS; dependent variable). The results are presented in Table 6. The factors found to be statistically significantly associated with caregivers’ fatigue, after multivariable analysis, were length of hospitalization (*p* = 0.047), as well as anxiety levels (*p* = 0.001) and insomnia levels (*p* = 0.003). More specifically, caregivers of patients hospitalized for 7–10 days reported significantly higher fatigue compared to those whose patients were hospitalized for only 1–3 days, with a regression coefficient β = 3.70 (95% CI: 0.05–7.45).

Finally, psychological factors were found to have a decisive impact: each one-point increase in the anxiety score (Zung) was associated with a 0.44-point increase in fatigue (95% CI: 0.21–0.67), while each one-point increase in the insomnia score (AIS) was associated with a 0.59-point increase in fatigue (95% CI: 0.20–0.98). These findings highlight that, beyond demographic and clinical characteristics, psychological burden and experiences related to caregivers’ daily life and environment significantly influence their levels of fatigue.

## 4. Discussion

In the present study, caregivers of hospitalized patients exhibited moderate fatigue, which demonstrated a positive correlation with anxiety and insomnia. Factors associated with caregivers’ fatigue included gender (female), length of hospitalization, experience of environmental stressors and financial stressors, and lack of familiarity with caregiving procedures in the hospital.

The fatigue experienced by caregivers appears to differ depending on the specific characteristics of the patient’s condition. Among caregivers of inpatients recovering from ischemic stroke, physical fatigue has been documented as severe, whereas psychological fatigue tends to be moderate. Furthermore, the extent of fatigue reported by caregivers in this population is significantly associated with the level of care dependency demonstrated by the stroke survivors [17]. Similarly, caregivers of patients with advanced-stage cancer undergoing radiotherapy reported significant fatigue-related difficulties both at baseline and at a six-month follow-up [18]. Furthermore, in a sample of 170 family caregivers of patients undergoing hemodialysis, the majority (88%) experienced moderate to severe fatigue. Caregiver fatigue emerged as a significant factor adversely affecting their quality of life [19]. An earlier study conducted by Choi et al. [20] (2014) examined caregivers of adult intensive care unit (ICU) survivors and reported that between 43% and 53% of caregivers experienced clinically significant fatigue at multiple time points, including during admission, two weeks post-admission, and at two and four months following ICU discharge. While caregiver fatigue has been studied in aforementioned specific populations (cancer patients, stroke or ICU survivors), there is comparatively less research focusing specifically on fatigue in caregivers of hospitalized patients in medical -surgical wards. This gap is often noted in the caregiving and health literature, highlighting the need for more focused research.

The findings of the present study indicate that, as caregivers’ anxiety and insomnia increased, fatigue levels also rose correspondingly. These findings are further supported by a study conducted in Korea by Kang et al. [11], which identified anxiety, depression, and daytime sleepiness as primary factors influencing fatigue among caregivers of patients with severe illnesses requiring long-term treatment. Similarly, a cross-sectional study in Taiwan involving 84 family members caring for patients with dementia reported a positive correlation between fatigue and anxiety [21]. Furthermore, a study conducted in Greece with a convenience sample of 291 caregivers attending health centers found that caregiver burden was positively associated with anxiety and stress [22]. Van der Velde et al. (2023) [23] found that caregiver strain and fatigue are independent predictors of anxiety in patients shortly after stroke. Fatigue, anxiety, and insomnia may result from the prolonged physical and emotional demands of caregiving.

According to the current results, a one-point increase in the anxiety score was associated with a 0.44-point increase in fatigue, while each one-point increase in the insomnia score was associated with a 0.59-point increase in fatigue. Fatigue, anxiety, and insomnia among caregivers of hospitalized patients interact in a cyclical manner, thereby exacerbating the overall burden experienced by caregivers. Fatigue stems from sustained caregiving efforts [2,22], while anxiety reflects the psychological stress inherent in caregiving responsibilities [8]. Insomnia often arises as a consequence of anxiety and disrupted sleep patterns intensifying the cumulative strain on caregivers [24]. Therefore, assessment of fatigue, anxiety, and insomnia as an interrelated triad among caregivers of hospitalized patients is essential given the synergistic effects these conditions exert on caregiver health.

Regarding demographics, female-gender was associated with caregivers’ fatigue. Conversely, among informal caregivers of hospitalized frail older patients, male gender was significantly associated with increased caregiver burden, in combination with prior caregiving experience, negative emotions, and maladaptive coping strategies [7]. However, evidence demonstrates that female caregivers bear a greater burden than their male counterparts, highlighting persistent gender disparities. In a relevant study among caregivers of cancer patients, being female increased the likelihood of experiencing fatigue and sleep disturbance [25]. Recent research suggests that female caregivers tend to experience higher levels of fatigue and emotional exhaustion compared to their male counterparts. Savla et al. [26] (2025) found that wives report greater caregiver overload and loneliness than husbands, with these factors being closely linked to fatigue. Qualitative research by Ramírez-Perdomo et al. [27] (2024) further highlights the gender-related dimensions of caregiving fatigue, with women describing their experiences in terms of emotional exhaustion and continuous vigilance. A recent study (2024) identified a gender bias in the perception of fatigue, revealing that, although women report higher levels of fatigue than men, their fatigue is often underestimated by others [28]. One proposed explanation for increased fatigue among female caregivers is the cumulative impact of physical, emotional, and social demands inherent in caregiving roles. Women often find caregiving tasks physically demanding, experience emotional stress from caring for loved ones, and manage additional family and household responsibilities, which together increase fatigue. However, it is important to note that many studies rely on proxy measures of fatigue, such as emotional exhaustion or caregiver burden, rather than standardized fatigue assessments.

Results also revealed that fatigue was significantly associated with hospitalizations lasting 7–10 days, a period during which the sustained demands of caregiving likely intensify caregiver strain. Shorter stays may not impose sufficient caregiving burden to cause marked fatigue, while longer hospitalizations might allow caregivers to develop coping strategies or receive additional support, thereby mitigating fatigue levels. This suggests that the 7–10-day timeframe represents a critical threshold for caregiver fatigue. In one relevant study, caregiver fatigue was associated with prolonged institutional care and worsened when patients did not return home, whereas hospital discharge was linked to improvement [20]. Ilaghi et al. (2024) [29] found that the duration of caregiving in patients with traumatic brain injury was a significant predictor of burden, suggesting a cumulative effect over time. A systematic review by Lindt et al. [30] (2020), exploring determinants of caregiver burden in Western countries, found that duration of caregiving was one of the most important predictors of caregiver burden. Extended caregiving hours reduce opportunities for rest, resulting in physical and emotional sustained exhaustion and diminished coping capacity [31,32,33].

Regarding perceptions of caregivers, the experience of environmental stressors and economic stressors, and unfamiliarity with the provision of care were associated with fatigue. Increased financial burden, stemming from extended weekly caregiving hours, financial insecurity, and related economic challenges, may contribute to greater caregiver fatigue. Caregivers frequently dedicate a substantial portion of their income and savings to support family members and may also face job loss, limited career opportunities, labor force withdrawal, or reduced working hours [2,4]. Astonishingly, one in six caregivers of individuals aged 50 and older report experience financial strain. The economic burden of unpaid family caregiving is considerable, with the estimated opportunity cost reaching up to USD 67 billion in lost earnings [4].

Environmental factors, such as thermal discomfort, inadequate lighting, and night-time noise consist major contributors to disturbed sleep and in turn to increased fatigue. Meanwhile, frequent night-time noise stemming from staff activity or medical check are associated with numerous wake-episodes and reduced sleep continuity [34]. A study in oncology showed that caregivers identify stressors during hospitalization including sleep deprivation, noise, and uncertainty that may contribute to psychological strain [35].

The finding that unfamiliarity with caregiving tasks is associated with fatigue underscores its significant contribution to caregiver exhaustion. When caregivers lack knowledge or experience in providing care, they often face increased stress and anxiety, which may lead to emotional and physical exhaustion. This lack of confidence may result in inefficient caregiving practices, longer time spent on tasks, and reduced opportunities for rest, all of which intensify fatigue. Caregivers must develop essential skills to effectively manage the daily demands of caregiving and alleviate perceived burden. The acquisition of such skills is critical for facilitating a successful transition into the caregiving role, as increased knowledge enhances adaptability and self-efficacy. While informal family caregivers develop expertise that facilitates decision making and role assertion, limited research explores the foundational knowledge and processes through which these skills are acquired and sustained over time, leaving critical questions unanswered [36].

Understanding these perceptions enables healthcare providers to identify caregivers’ modifiable factors that affect patients’ recovery. Moreover, integrating perceptions into clinical care supports more patient-centered, holistic interventions that enhance satisfaction, adherence, and long-term adjustment to the disease [37]. With the rising prevalence of chronic illnesses, caregiving responsibilities frequently become prolonged, resulting in increased incidences of sleeplessness, fatigue, and emotional distress among family caregivers [20].

Last but not least, it is important to acknowledge confounding factors that may influence the relationship among the triad of fatigue, anxiety, and insomnia in caregivers of hospitalized patients. Care-related factors, such as the number of hours spent per day at the hospital and the patient’s level of dependency or illness severity, may play a significant role. Furthermore, psychosocial factors, including perceived social support, coping strategies, and mental health history, may confound the impact of this triad. Moreover, hospital-related factors such as private versus public institutions, availability of social services, distance from the hospital, and the number of healthcare providers may further contribute to caregiver fatigue. These confounding variables must be controlled to accurately assess the associations between fatigue and caregiver characteristics, as well as the triad symptoms of fatigue, insomnia, and anxiety. In the present study, caregivers over 60 years old accounted for 53.5%, and female caregivers comprised 64.8%. These participants may have reduced physical strength, which could contribute to increased fatigue.

### Limitations of This Study

Our study has several limitations. Given its cross-sectional design, it is not possible to infer the directionality or causality of the observed associations. A longitudinal design with repeated measures at 6–12 months would better assess potential changes in fatigue, anxiety, and insomnia over time. Additionally, the use of non-probability (convenience) sampling limits the representativeness of the sample to all caregivers of hospitalized patients in Greece, thereby restricting the generalizability of the findings. Participants were recruited from a single general public hospital, which may introduce bias. Expanding the sample size and conducting multi-institutional and multi-regional studies would enhance the validation of the results. Furthermore, a larger sample may reveal additional statistically significant correlations. The discussion may also have been influenced by the search strategy, which included only studies published in English and indexed in electronic databases. Finally, further insights could be gained by exploring additional parameters, such as (a) evaluation during the pre-hospital phase and (b) assessment following hospital discharge.

## 5. Conclusions

In the present study caregivers of hospitalized patients had moderate fatigue and insomnia as well as low to moderate anxiety. As caregivers’ anxiety and insomnia increase, fatigue then also increases. Factors associated with caregivers’ fatigue levels were as follows: gender (female), length of hospitalization, experience of environmental stressors and financial stressors, and unfamiliarity with the provision of care while at the hospital. A better understanding of these factors can enhance the identification, outreach, and support of caregivers. Future prospective studies should explore interventions aimed at reducing fatigue among caregivers.

A comprehensive understanding of the triad of fatigue, anxiety, and insomnia is crucial for informing the design of holistic interventions aimed at mitigating the multifactorial burden experienced by caregivers.

## Figures and Tables

**Table 1:** Basic characteristics of caregivers (*n* = 142).

	*n* (%)
**Caregivers**	
Spouse	92 (64.8%)
Child	50 (35.2%)
Gender	
Male	50 (35.2%)
Female	92 (64.8%)
Age	
40–50	30 (21.1%)
51–60	36 (25.3%)
61–70	42 (29.6%)
>70	34 (24.0%)
Marital status	
Married	90 (63.4%)
Single	34 (23.9%)
Divorced	16 (11.3%)
Widowed	2 (1.4%)
Educational level	
Primary	30 (21.1%)
Secondary	54 (38.1%)
Tertiary	58 (40.8%)
Occupation	
Household	12 (8.4%)
Public sector employee	40 (28.2%)
Private sector employee	47 (33.1%)
Self-employed	17 (12.0%)
Retired	26 (18.3%)
Place of residence	
Attica	67 (47.2%)
Prefecture capital	64 (45.1%)
Rural	11 (7.7%)
Duration of patient’s hospitalization	
1–3 days	32 (22.6%)
4–6 days	41 (28.9%)
7–10 days	34 (23.9%)
10 days	35 (24.6%)

**Table 2:** Caregivers’ perceptions (*n* = 142).

	*n* (%)
Self-reported level of information about patient’s health status	
Very well informed	44 (31.0%)
Sufficiently informed	68 (47.9%)
Slightly informed	29 (20.4%)
Not at all informed	1 (0.7%)
Emotional support to patient after hospital discharge	
Very much	81 (57.1%)
Quite a lot	48 (33.8%)
A little	11 (7.7%)
Not at all	2 (1.4%)
Environmental/hospital stressors	
Yes	111 (78.2%)
No	31 (21.8%)
Financial stressors due to patient’s illness	
Very much	41 (28.9%)
Quite a lot	37 (26.1%)
A little	34 (23.9%)
Not at all	30 (21.1%)
Unfamiliarity with care provision during hospitalization	
Very much	52 (36.6%)
Quite a lot	45 (31.7%)
A little	35 (24.7%)
Not at all	10 (7.0%)
Need for communication with nurses	
Yes	131 (92.3%)
No	11 (7.7%)

**Table 3:** Description of caregivers’ fatigue, anxiety, and insomnia (*n* = 142).

	Cronbach’s Alpha	Mean (SD)	Median (IQR)
Fatigue (FAS) (Range 10–50)	0.730	25.9 (9.3)	25 (19–31)
Anxiety (SAS) (Range 20–80)	0.864	38.1 (9.0)	37 (32–45)
Insomnia (AIS) (Range 0–24)	0.881	7.6 (4.7)	7 (4–10)
	*n* (%)		
Insomnia Score ≥ 6	92 (64.8%)		

SD: Standard Deviation, IQR: Interquartile Range.

**Table 4:** Correlation of fatigue with anxiety and insomnia.

	Fatigue (FAS)	
	Spearman’s Rho	*p*-Value
Anxiety (Score Zung)	0.713	0.001
Insomnia (Score AIS)	0.671	0.001

**Table 5:** Association of fatigue with caregivers’ characteristics.

	Fatigue (FAS)		
	Mean (SD)	Median (IQR)	*p*-Value *
Relationship to patient			0.575
Spouse	26.4 (11.3)	24 (19–32)	
Child	24.7 (7.4)	23 (19–30.5)	
Gender			0.001
Male	21.7 (6.5)	21 (16–26)	
Female	27.4 (10.0)	26 (21–33)	
Age			0.840
40–50	27.3 (13.1)	27 (19–32)	
51–60	24.2 (7.2)	22 (19.5–28.5)	
61–70	25.1 (7.1)	24.5 (20–28)	
>70	25.3 (9.0)	22.5 (18–33)	
Marital status			0.467
Married	24.7 (7.8)	23 (19–29)	
Single	28.1 (13.2)	25.5 (19–33)	
Divorced/Widowed	23.8 (6.5)	25 (19–29)	
Educational level			0.200
Primary	26.4 (7.7)	25 (21–32)	
Secondary	24.0 (7.0)	23 (18–29)	
Tertiary	26.4 (16.0)	20 (16–32)	
Occupation			0.518
Household	25.3 (5.9)	26 (21.5–29)	
Public sector employee	25.8 (11.7)	24 (19–30.5)	
Private sector employee	25.9 (8.1)	24 (21–32)	
Self-employed	22.4 (7.3)	20 (18–26)	
Retired	26.0 (9.6)	25 (18–36)	
Place of residence			0.224
Attica	26.8 (10.7)	25 (21–32)	
Regional capital	24.6 (8.2)	23 (18–31)	
Rural area	22.1 (4.9)	21 (18–27)	
Length of hospitalization in days			0.013
1–3	24.4 (6.8)	23 (19–28.5)	
4–6	23.3 (11.9)	21 (17–26)	
7–10	27.5 (7.6)	27 (21–32)	
10	26.9 (8.9)	27 (19–35)	
Informed about the patient’s health status			0.348
Very much	24.8 (7.3)	23.5 (19–29)	
Quite	24.4 (7.8)	23 (18.5–30.5)	
Little/Not at all	28.6 (13.6)	26.5 (19–33)	
Support for the patient after discharge			0.199
Very much	24.9 (7.5)	24 (19–29)	
Quite	27.3 (11.8)	25.5 (19–32.5)	
Little/Not at all	22.1 (8.0)	20 (16–27)	
Experience of environmental stressors			0.045
Yes	26.1 (9.5)	24 (19–32)	
No	23.2 (8.4)	21 (17–26)	
Financial stressors due to patient’s health			0.001
Very much	28.4 (8.0)	29 (23–33)	
Quite	25.1 (6.4)	23 (21–28)	
Little	25.1 (7.3)	23 (19–32)	
None	22.2 (14.0)	18.5 (15–24)	
Unfamiliarity with care during hospitalization			0.001
Very much	28.6 (6.8)	28 (24–32)	
Quite	25.7 (7.3)	24 (19–31.5)	
Little/None	22.0 (12.1)	19 (16–22)	
Need for communication with nurses			0.948
Yes	25.1 (7.7)	24 (19–31)	
No	29.8 (20.7)	22 (18–31)	

* was calculated with non-parametric tests of Mann–Whitney and Kruskal–Wallis.

**Table 6:** Estimation of the impact of caregivers’ characteristics on fatigue (FAS).

	Fatigue (FAS) β Coef (95% CI)	*p*-Value
Gender		
Male	Ref. Cat.	
Female	2.37 (−0.56–5.29)	0.112
Length of hospitalization in days		
1–3	Ref. Cat.	
4–6	2.35 (−1.37–6.08)	0.214
7–10	3.70 (0.05–7.45)	0.047
>10	2.65 (−1.22–6.51)	0.178
Environmental stressors		
Yes	Ref. Cat.	
No	−3.02 (−6.62–0.57)	0.099
Financial stressors due to patient’s health		
Very much	Ref. Cat.	
Quite a lot	−0.97 (−4.49–2.55)	0.587
A little	0.22 (−3.64–4.08)	0.909
Not at all	−0.19 (−4.48–4.10)	0.929
Unfamiliarity with care during hospitalization		
Very much	Ref. Cat.	
Quite a lot	−2.80 (−6.18–0.58)	0.103
A little/Not at all	−1.70 (−5.48–2.08)	0.375
Anxiety (Zung)	0.44 (0.21–0.67)	0.001
Insomnia (AIS)	0.59 (0.20–0.98)	0.003

CI: Confidence Interval, Ref. Cat.: Reference Category.

## Data Availability

The original contributions presented in this study are included in the article. Further inquiries can be directed to the corresponding author.

## References

[1] Canetta C., Accordino S., La Boria E., Arosio G., Cacco S., Formagnana P., Masotti M., Provini S., Passera S., Viganò G. (2024). Effects of a medical admission unit on in-hospital patient flow and clinical outcomes. Eur. J. Intern. Med..

[2] Stavrianou A., Kafkia T., Mantoudi A., Minasidou E., Konstantinidou A., Sapountzi-Krepia D., Dimitriadou A. (2018). Informal Caregivers in Greek Hospitals: A Unique Phenomenon of a Health System in Financial Crisis. Mater. Sociomed..

[3] Pozet A., Darnis S., Bonnet M., Meurisse A., Dabakuyo-Yonli T.S., Lejeune C., Fagnoni P., Gaimard M., Manckoundia P., Quibel C. (2023). Quality of Life and Needs in Caregivers: Results from the Prospective Multicentric Open-Label Randomized Study of Informal Caregivers of Elderly Patients. Int. J. Public Health.

[4] Wagle S., Yang S., Osei E.A., Katare B., Lalani N. (2024). Caregiving Intensity, Duration, and Subjective Financial Well-Being Among Rural Informal Caregivers of Older Adults with Chronic Illnesses or Disabilities. Healthcare.

[5] Lv H., Yang S., Zhang Y., Wang Y., Zhang L., Wang J., Jiang H. (2025). Caregiver Burden and Associated Factors Among Informal Caregivers of Hospitalized Elderly Patients in China: A Latent Profile Analysis. Risk Manag. Healthc. Policy.

[6] Germain N., Jémus-Gonzalez E., Couture V., Côté É., Morin M., Toulouse-Fournier A., Bert L., Giguère R., Sinha S., Sourial N. (2024). Network of Canadian Emergency Researchers. Caregivers’ burden of care during emergency department care transitions among older adults: A mixed methods cohort study. BMC Geriatr..

[7] Lan X., Wu Q., Chen X., Jin S., Yi B. (2021). Caregiver burden among informal caregivers of hospitalized patients with frailty: A cross-sectional survey. Geriatr. Nurs..

[8] Cejalvo E., Martí-Vilar M., Merino-Soto C., Aguirre-Morales M.T. (2021). Caregiving Role and Psychosocial and Individual Factors: A Systematic Review. Healthcare.

[9] Lenz E.R., Pugh L.C., Milligan R.A., Gift A., Suppe F. (1997). The middle-range theory of unpleasant symptoms: An update. ANS Adv. Nurs. Sci..

[10] Pearlin L.I., Mullan J.T., Semple S.J., Skaff M.M. (1990). Caregiving and the stress process: An overview of concepts and their measures. Gerontologist.

[11] Kang S.G., Song S.W., Kim S.H., Kang Y.J., Kim Y.R., Eun Y. (2020). Fatigue and Mental Status of Caregivers of Severely Chronically Ill Patients. Pain Res. Manag..

[12] Song S.W., Kang S., Kim K.S., Kim M.J., Kim K.M., Cho D.Y., Kim Y.S., Joo N.S., Kim K.N. (2018). Reliability and Validity of the Korean Version of the Multidimensional Fatigue Inventory (MFI-20): A Multicenter, Cross-Sectional Study. Pain Res. Manag..

[13] Althubaiti A. (2022). Sample size determination: A practical guide for health researchers. J. Gen. Fam. Med..

[14] Alikari V., Fradelos E., Sachlas A., Panoutsopoulos G., Lavdaniti M., Palla P., Lappa T., Giatrakou S., Stathoulis J., Babatsikou F. (2016). Reliability and validity of the Greek version of “The Fatigue Assessment Scale”. Arch. Hell. Med..

[15] Samakouri M., Bouhos G., Kadoglou M., Giantzelidou A., Tsolaki K., LIvaditis M. (2012). Standardization of the Greek version of Zung’s Self-Ratting Anxiety Scale. Psychiatriki.

[16] Soldatos C.R., Dikeos D.G., Paparrigopoulos T.J. (2000). Athens Insomnia Scale: Validation of an instrument based on ICD-10 criteria. J. Psychosom. Res..

[17] Tu S., Deng Z., Li S., Wang J., Yang R., Zhao L. (2025). Care dependence and caregiver fatigue in ischemic stroke survivors: A cross-sectional study. BMC Nurs..

[18] Clark M.M., Atherton P.J., Lapid M.I., Rausch S.M., Frost M.H., Cheville A.L., Hanson J.M., Garces Y.I., Brown P.D., Sloan J.A. (2014). Caregivers of patients with cancer fatigue: A high level of symptom burden. Am. J. Hosp. Palliat. Care..

[19] Akbari R., Farsi Z., Sajadi S.A. (2023). Relationship between fatigue and quality of life and related factors in family caregivers of patients on hemodialysis. BMC Psychiatry.

[20] Choi J., Tate J.A., Hoffman L.A., Schulz R., Ren D., Donahoe M.P., Given B.A., Sherwood P.R. (2014). Fatigue in family caregivers of adult intensive care unit survivors. J. Pain. Symptom Manag..

[21] Jiang J.L., Chang S.L., Wang K.C., Ma Y.C. (2025). Relationship between anxiety and fatigue in dementia family caregivers: Hope as a mediator. BMC Nurs..

[22] Albani E.N., Toska A., Togas C., Rigatos S., Vus V., Fradelos E.C., Tzenalis A., Saridi M. (2024). Burden of Caregivers of Patients with Chronic Diseases in Primary Health Care: A Cross-Sectional Study in Greece. Nurs. Rep..

[23] van der Velde M.Y., Aerden L.A.M., van Oort A., Bodde K., Rambaran Mishre R., Oosterveer D.M. (2023). Caregiver strain and fatigue are independent determinants of anxiety among patients early after stroke. Neuropsychol. Rehabil..

[24] Fernández-Puerta L., Prados G., Quiñoz-Gallardo M.D., Vellido-González D., González-Guerrero M.L., Rivas-Campos A., Jiménez-Mejías E. (2023). Insomnia Symptoms and Associated Factors in Caregivers of Adult Hospitalized Patients. Healthcare.

[25] Johansen S., Cvancarova M., Ruland C. (2018). The Effect of Cancer Patients’ and Their Family Caregivers’ Physical and Emotional Symptoms on Caregiver Burden. Cancer Nurs..

[26] Savla J., Roberto K.A., Fontaine L.A. (2025). Gender differences in spousal caregiver strain and paid service use among dementia caregivers in rural Appalachia. Front. Public Health.

[27] Ramírez-Perdomo C.A., Cantillo-Medina C.P., Perdomo-Romero A.Y. (2024). Time for Care: Male and Female Voices Based on Their Caregiving Experiences. Healthcare.

[28] Stosic M.D., Flynn-Evans E.E., Duenas J., Ruben M.A. (2024). Gender Bias in the Perception of Others’ Fatigue: Women Report More Fatigue Than Men But Have Their Fatigue Underestimated by Others. Sex Roles.

[29] Ilaghi M., Gharib F., Pirani A., Vahabie A.H., Grafman J., Shariat S.V., Shariati B., Jahanbakhshi A., Mirfazeli F.S. (2024). The burden of traumatic brain injury on caregivers: Exploring the predictive factors in a multi-centric study. BMC Psychol..

[30] Lindt N., van Berkel J., Mulder B.C. (2020). Determinants of overburdening among informal carers: A systematic review. BMC Geriatr..

[31] Gérain P., Zech E. (2019). Informal Caregiver Burnout? Development of a Theoretical Framework to Understand the Impact of Caregiving. Front. Psychol..

[32] Liu Z., Heffernan C., Tan J. (2020). Caregiver burden: A concept analysis. Int. J. Nurs. Sci..

[33] Choi J.Y., Lee S.H., Yu S. (2024). Exploring Factors Influencing Caregiver Burden: A Systematic Review of Family Caregivers of Older Adults with Chronic Illness in Local Communities. Healthcare.

[34] Yang E., Ismail A., Kim Y., Erdogmus E., Boron J., Goldstein F., DuBose J., Zimring C. (2022). Multidimensional Environmental Factors and Sleep Health for Aging Adults: A Focused Narrative Review. Int. J. Environ. Res. Public. Health.

[35] Abuatiq A., Brown R., Wolles B., Randall R. (2020). Perceptions of Stress: Patient and Caregiver Experiences with Stressors During Hospitalization. Clin. J. Oncol. Nurs..

[36] Leocadie M.C., Morvillers J.M., Pautex S., Rothan-Tondeur M. (2020). Characteristics of the skills of caregivers of people with dementia: Observational study. BMC Fam. Pract..

[37] Polikandrioti M. (2021). Patient Perceptions and Quality of Life in Pacemaker Recipients. J. Innov. Card. Rhythm. Manag..

